# Coordinated topoisomerase function shapes the fluoroquinolone response of *Chlamydia trachomatis*

**DOI:** 10.1128/spectrum.02206-26

**Published:** 2026-08-27

**Authors:** Andrew Terrebonne, Caitlynn Diggs, Scot P. Ouellette, Leiqiong Gao, Yuk-Ching Tse-Dinh, Li Shen

**Affiliations:** 1 Department of Microbiology, Immunology, and Parasitology, Louisiana State University Health Sciences Center 547965, New Orleans, Louisiana, USA; 2 Department of Pathology, Microbiology, and Immunology, University of Nebraska Medical Center 12284, Omaha, Nebraska, USA; 3 Biomolecular Sciences Institute, Department of Chemistry and Biochemistry, Florida International University 5450, Miami, Florida, USA; University of Louisville, Louisville, Kentucky, USA

**Keywords:** DNA gyrase, topA, DNA supercoiling, *Chlamydia trachomatis*, fluoroquinolone moxifloxacin, persistent form, DNA replication, transcription, antibiotic tolerance, stress adaptation

## Abstract

DNA supercoiling is essential for the developmental cycle of *Chlamydia trachomatis*, yet its role in shaping antibiotic responses remains poorly understood. We investigated how the fluoroquinolone moxifloxacin (Mox), a potent DNA gyrase inhibitor, affects *C. trachomatis* by examining developmental progression, DNA replication, topoisomerase expression, and transcriptional response in select genes. Early Mox exposure completely halted bacterial growth, whereas treatment during mid-developmental cycle produced small inclusions with enlarged bacterial forms and abolished formation of infectious progeny. These outcomes coincided with inhibition of DNA replication, apparent loss of detectable DNA gyrase, and repression of *ompA* and *omcB* transcription, while the expression of the *groESL1* operon was preserved or elevated. Mox also elicited the downregulation of topoisomerase I (TopA), consistent with compensatory rebalancing of DNA supercoiling. Together, these data demonstrate that Mox exerts differential effects across the developmental stages and that disrupted gyrase-TopA coordination drives chlamydial developmental arrest and a noncanonical, DNA replication-limited persistent phenotype

**IMPORTANCE:**

*C. trachomatis*, a clinically significant obligate intracellular bacterial pathogen, can persist under antimicrobial pressure, complicating treatment strategy. This study links supercoiling homeostasis to fluoroquinolone tolerance, providing mechanistic insights into chlamydial responses to topological stress and identifying potential targets to overcome noncanonical persist state.

## INTRODUCTION

*Chlamydia trachomatis* (Ctr) is a Gram-negative, obligate intracellular pathogen and the leading cause of bacterial sexually transmitted infections (STIs) worldwide (1–3). Many infections are asymptomatic, and untreated and/or chronic Ctr infection can lead to severe sequelae, including pelvic inflammatory disease, ectopic pregnancy, infertility, epididymitis, and reactive arthritis. Neonates born to mothers with untreated Ctr genital infections can develop severe conjunctivitis and pneumonia. Epidemiological studies further link Ctr infection to increased susceptibility to other sexually transmitted pathogens and to long-term complications such as cervical and ovarian cancers (4). Despite the availability of effective antibiotics, persistent or recurrent infections remain a clinical challenge (3, 5, 6), and the underlying mechanisms that enable Ctr to survive antimicrobial pressure are not fully understood.

Ctr pathogenesis is closely tied to its unique biphasic developmental cycle (7, 8). Following entry into host mucosal epithelial cells, the infectious elementary body (EB) quickly decondenses its chromatin and differentiates into a noninfectious yet replicative reticulate body (RB) in a membrane-bound inclusion. During the mid-developmental cycle (~8–16 hours post-infection [hpi]), RBs undergo exponential replication and expansion of the inclusion. Subsequently, RBs asynchronously initiate secondary differentiation into EBs. The developmental cycle is typically completed within 48–72 h, culminating in host-cell lysis or extrusion of the inclusion and release of infectious EB progeny, which then initiate new rounds of infection. Under stress conditions, including nutrient deprivation (9), interferon-γ exposure (10, 11), and certain antibiotic treatments (11–13), Ctr can enter a persistent state, which is characterized by morphologically aberrant persistent forms that remain viable but fail to produce infectious progeny in culture. Because persistence is typically reversible upon stress removal, clinical Ctr persistence is increasingly recognized as a major contributor to chronic infection and treatment failure (14–17).

Progression through the Ctr developmental cycle relies on timed waves of transcription modulated by sigma factors (18–20), transcriptional regulators (21–24), and DNA topology (25–27). DNA gyrase introduces negative supercoils into the bacterial chromosome, while TopA counterbalances this activity by relaxing negatively supercoiled DNA, together maintaining supercoiling homeostasis (28–31). Quinolones, classified into four generations, are broad-spectrum bactericidal antibiotics that exert toxicity by targeting DNA gyrase and/or Topo IV (32–34). However, their widespread clinical use has driven the emergence of resistance (34–36). Levofloxacin (a third-generation fluoroquinolone) and moxifloxacin (Mox) (a fourth-generation fluoroquinolone) are currently listed as alternative therapies for STI causing Ctr and *Mycoplasma genitalium*, respectively (3). Although fluoroquinolone-resistant Ctr clinical isolates remain rare, recent reports raise concerns that resistance may be actively emerging (16, 37). Experimental work indicates that exposure of Ctr to fluoroquinolones can select resistance-associated mutations (38, 39) and/or induce a state of metabolic dormancy or persistence (13, 40).

Recent findings highlight the importance of balanced gyrase and TopA activity for chlamydial development and antibiotic response (41, 42). Indeed, CRISPR interference (CRISPRi)-mediated repression of *topA* disrupts supercoiling homeostasis, arrests development, and sensitizes Ctr to fluoroquinolones (41, 42). However, how lethal fluoroquinolone exposure perturbs DNA supercoiling and topoisomerase expression during infection, and how these changes shape downstream Ctr adaptive responses, remains unresolved.

In this study, Mox was used as a potent, well-characterized gyrase inhibitor to probe the mechanistic consequences of impaired DNA supercoiling on chlamydial development, a process inherently marked by stage-specific variations in baseline supercoiling levels (25–27). We characterize Mox-induced changes in chlamydial developmental progression, morphology, DNA replication, topoisomerase expression, and promoter-specific transcription in selective genes. These findings define how supercoiling dynamics shape the chlamydial response to Mox-mediated topological stress, with imbalance in this system driving a replication-limited, noncanonical persistent state.

## RESULTS

### Mox exerts developmental stage-dependent effects on Ctr

To assess how developmental timing influences the susceptibility of Ctr to Mox, HeLa 229 cells (HeLa hereafter) were infected with a green fluorescence protein (GFP) expressing strain (L2/P*_ompA_*-GFP) (Table S1) (43), followed by exposure to increasing concentrations of Mox at 0, 8, 16, or 24 hpi. These time points correspond to published variations in Ctr DNA supercoiling, which is low shortly after infection, increases during mid-cycle, peaks at approximately 24 hpi, and declines during the late stage (25, 27)

**Fig 1:**
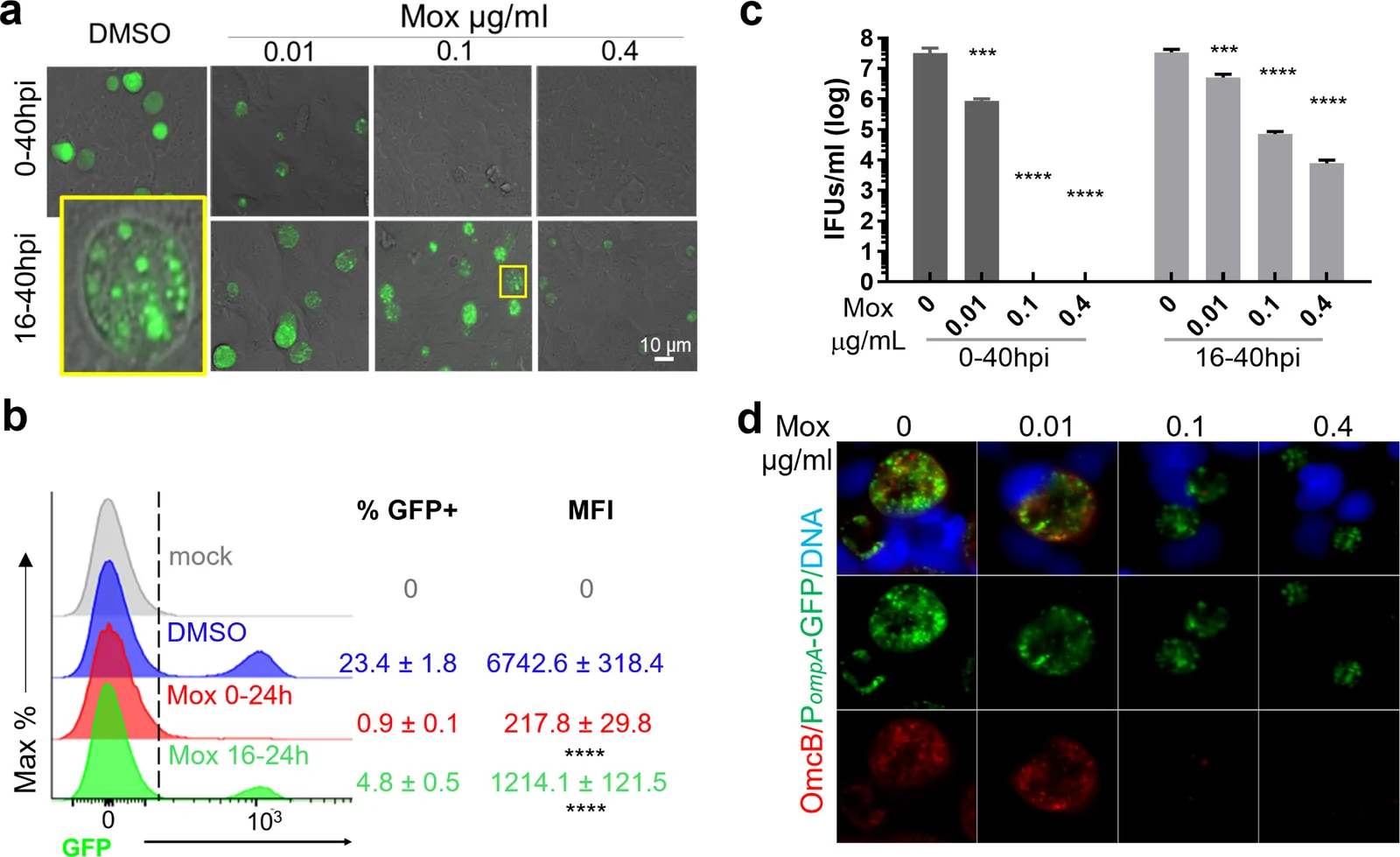
Time- and dose-dependent effects of Mox on *C. trachomatis* growth. (**a**) Live-cell fluorescence micrographs of Ctr-infected cells. L2/P*_ompA_*-GFP-infected cultures were exposed to Mox or DMSO (control) starting at 0 or 16 hpi and imaged at 40 hpi. Inset shows magnified representative inclusions containing enlarged RB-like forms. Scale bar, 10 µm. (**b**) Flow-cytometric quantification of GFP^+^ cells (%) and mean fluorescence intensity (MFI). Cultures exposed to Mox at 0.1 µg/mL for 24 h (0–24 hpi) or 8 h (16–24 hpi) were fixed at 24 hpi and subjected to flow cytometry. Mock-infected cells and DMSO-treated infected cells were used as controls. Data represent means ± standard deviations (SD) from three biological replicates, each measured in technical triplicate. Statistical comparisons of MFI between exposed samples and DMSO control were made using one-way ANOVA with Tukey’s post hoc test. ***P < 0.0001. (**c**) Enumeration of infectious EB yields by inclusion-forming unit (IFU) assay at 40 hpi. The IFU values represent means ± SD from three biological replicates. Statistical significance was assessed by one-way ANOVA with Tukey’s post hoc test. ***P < 0.001; ****P < 0.0001. (**d**) Immunofluorescence micrograph of Ctr-infected cells. Fixed cells at 40 hpi were stained for OmcB (red; anti-OmcB antibody) and cellular and bacterial genomic DNA (blue; DAPI). GFP-expressing Ctr organisms were green. Scale bar, 10 µm.

Mox exposure initiated at 0 or 8 hpi completely prevented inclusion formation, confirming high sensitivity during early development. Treatment initiated during mid-development (16 hpi) produced significantly smaller inclusions containing enlarged RB-like forms (Fig. 1a). This was accompanied by a marked reduction in P*_ompA_*-GFP reporter levels, infectious EB progeny, and the expression of the late-stage EB-specific outer membrane protein OmcB (Fig. 1b through d). The appearance of the enlarged RBs is consistent with an immediate disruption of normal developmental progression. In contrast, the addition of Mox at a late stage (24 hpi) resulted in only mild developmental defects.

To assess recovery potential, Mox-containing medium was removed from infected cultures at 48 hpi. Cells continued incubating in drug-free medium for an additional 48 h in the absence of cycloheximide to preserve host cell metabolic defenses. Under these conditions, inclusion expansion resumed slowly and was accompanied by a modest (~4-fold) increase in EB production alongside fewer inclusions with typical morphology (Fig. S1). These observations suggest that while many inclusions may be damaged over time, a subset of organisms persists and retains the capacity to re-enter productive development following drug withdrawal.

These findings indicate that Mox exerts stage-dependent effects across the chlamydial developmental cycle, with the greatest disruption occurring during middevelopmental cycle. Although a large fraction of inclusions appears non-viable, a small subset of organisms remains capable of recovery after drug removal.

### Mox inhibits chromosomal DNA replication during the chlamydial exponential phase

**Fig 2:**
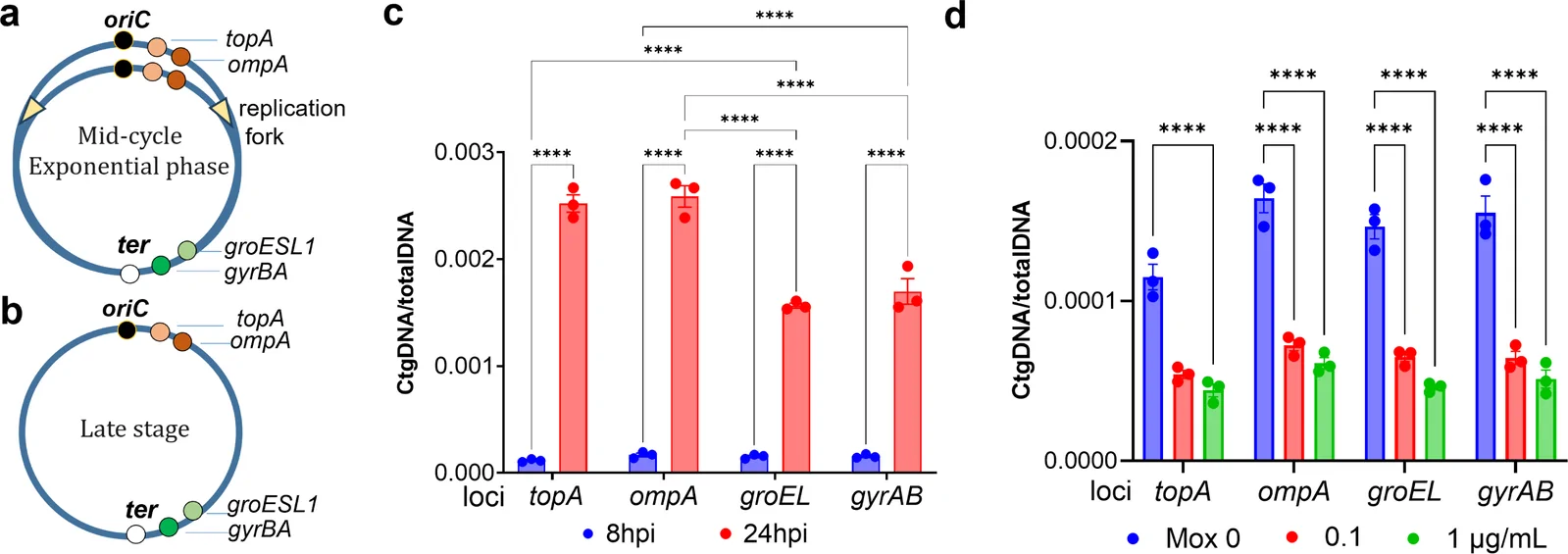
Effect of Mox on DNA replication in Ctr. DNA extracted from L2/434/Bu infected cells was used to quantify CtgDNA by qPCR with gene-specific primers. (**a and b**) Scheme of chromosomal loci proximal to the *oriC* and *ter* at exponential phase (mid-developmental cycle) (**a**) compared to late stage (**b**). Not to scale. (**c**) Quantitative changes in CtgDNA contents from 8 to 24 hpi. (**d**) Effect of Mox on CtgDNA contents during 8 to 24h. CtgDNA was normalized to total gDNA in respective samples. Data represent mean ± SD from three independent biological replicates. Statistical significance was determined using two-way ANOVA with the Tukey’s post hoc analysis; *P < 0.05, ***P  < 0.001, ****P <  0.0001.

To determine whether growth inhibition reflects impaired DNA replication, chlamydial genomic DNA (CtgDNA) accumulation was quantified by quantitative polymerase chain reaction (qPCR). Primer sets targeting loci proximal to the origin of replication (*oriC*) (e.g., *topA*, *ompA*) and the terminus (*ter*) (*groEL1*, *gyrBA*) were used (Fig. 2a and b; Table S2), enabling the assessment of replication activity and progression across the chromosome.

In unexposed cultures, CtgDNA increased steadily from 8 to 24 hpi, with relative enrichment of *oriC*-proximal loci (Fig. 2c), reflecting ongoing initiation and fork progression. Exposure to Mox (0.1 or 1 µg/mL) beginning at 8 hpi resulted in a marked reduction of CtgDNA at both *oriC*- and *ter*-proximal loci (Fig. 2d), indicating reduced DNA replication. A similar effect was observed when Mox was applied from 16 to 24 hpi (Fig. S2). Prolonged exposure (16–40 hpi) led to a further decline in CtgDNA, supporting sustained inhibition of replication.

These results suggest that DNA replication is particularly sensitive to Mox during the exponential phase in which RBs actively multiply, and the needs for negative supercoiling are likely elevated.

### Topoisomerase gene expression responds rapidly and differentially to Mox exposure

**Fig 3:**
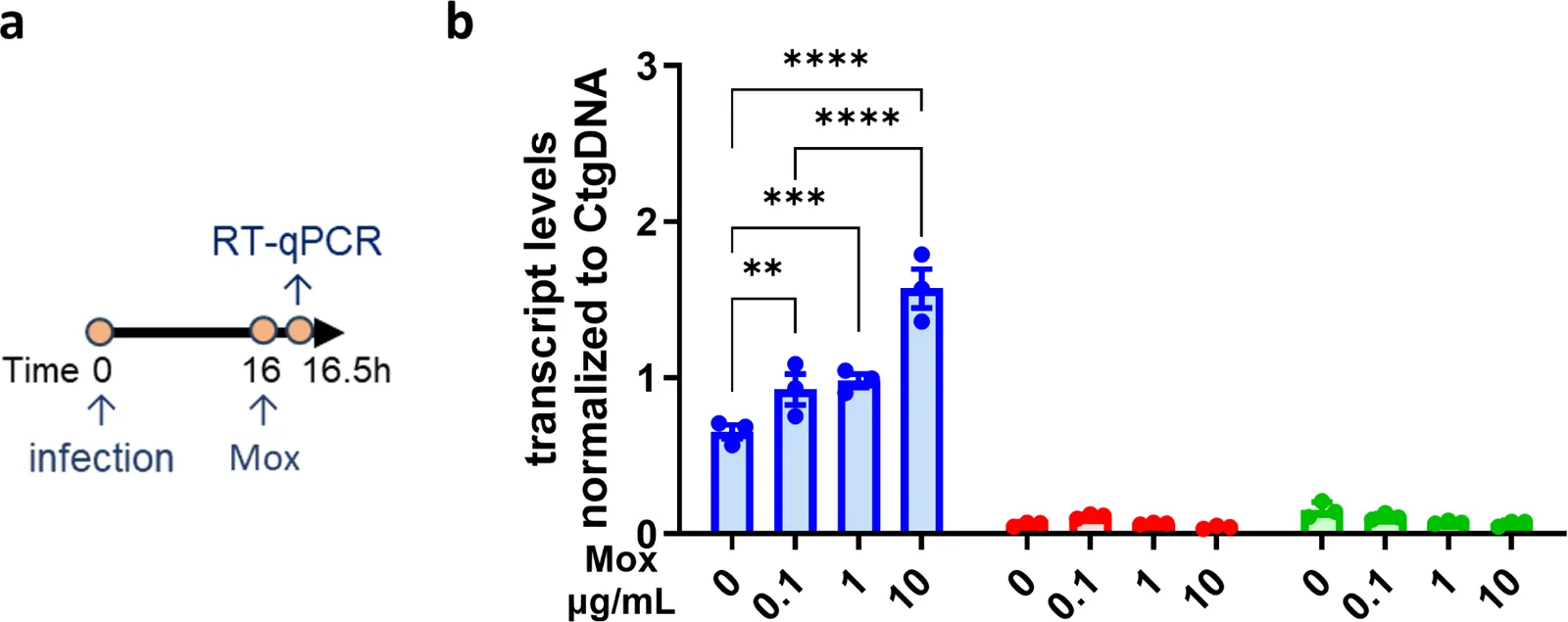
Mox exposure elicits transcriptional responses differently in topoisomerase genes. (**a**) Schematic diagram of the experimental procedure. L2/434/Bu cultures were exposed to Mox starting at 16 to 16.5 hpi. Unexposed, DMSO-treated cultures were used as controls. (**b**) Changes in relative transcript levels of genes as indicated following exposure to Mox (at 0, 0.1, 1, and 10 µg/mL). Quantified transcripts by RT-qPCR were normalized to the CtgDNA value from respective samples by qPCR with the same primer sets. Data represent means ± SD from three independent biological replicates. Statistical significance was determined by two-way ANOVA with Tukey’s post hoc test. *P* < 0.05, ***P < 0.001; ****P < 0.0001.

The study of gene responses to supercoiling variations is often conducted by using a wild-type strain and inducing a rapid change in supercoiling with topoisomerase-inhibiting antibiotics (25, 28, 44). The same approach was employed to assess transcription responses of topoisomerase genes to Mox during the mid-developmental cycle. Wild-type strain L2/434/Bu (Table S1) cultures were exposed to increasing concentrations of Mox for 30 min (16–16.5 hpi) (Fig. 3a), and transcript levels of *gyrAB* (encoding DNA gyrase), *parCE* (encoding Topo IV), and *topA* (encoding TopA) were quantified by RT-qPCR.

Mox exposure resulted in a rapid, dose-dependent increase in *gyrAB* transcript levels (Fig. 3b), indicating stimulation of *gyrAB* expression under conditions of reduced negative supercoiling. By contrast, *topA* and *parCE* transcript levels showed little change. These results demonstrate preferential induction of the DNA gyrase genes following Mox exposure and are consistent with the role of DNA gyrase in response to perturbations in supercoiling during the mid-developmental cycle.

### Mox alters detectable GyrA protein independently of transcriptional changes

Transcript level changes alone could not explain the severe phenotypic defects observed. To assess gyrase expression at the protein level, the levels of GyrA, the catalytic subunit of DNA gyrase, were analyzed in whole cell lysates by immunoblotting. A rabbit polyclonal anti-GyrA antibody was generated against the C-terminal region (amino acid residues 818–836). Chlamydial Hsp60 and host GAPDH served as bacterial and host protein controls, respectively, for normalization and comparison of GyrA levels.

**Fig 4:**
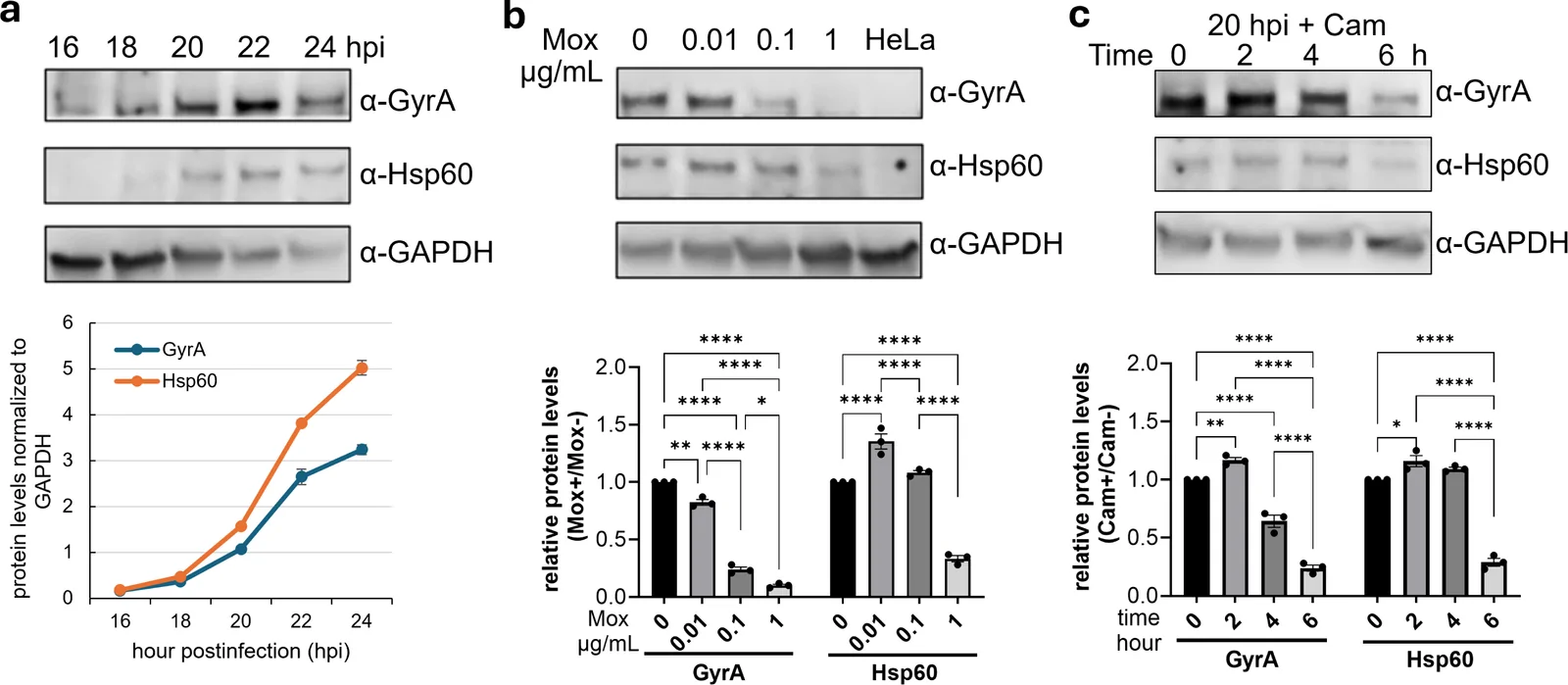
Mox reduces detectable GyrA levels in lysates from infected cells. (**a**) Trends in GyrA and Hsp60 accumulation over the indicated time course. Band intensities were normalized to GAPDH (*n = 3*). Mean ± SD values of normalized chlamydial proteins are plotted with time on the *x*-axis and relative protein levels on the *y*-axis. (**b**) Immunoblot analysis of detectable GyrA levels following Mox exposure for 6 h. Ctr L2/434/Bu-infected cells or mock-infected cells (as controls) were treated with increasing concentrations of Mox starting at 16 hpi and harvested at 22 hpi. Relative protein levels (GyrA/GAPDH or Hsp60/GAPDH) were normalized to the corresponding untreated control (set to 1). (**c**) Analysis of GyrA levels under translational arrest. Infected cells were treated with chloramphenicol (60 µg/mL) at 20 hpi and incubated for an additional 2, 4, or 6 h prior to analysis. Relative protein levels were normalized to the corresponding non-treated control (set to 1). Data are presented as mean ± SD from three independent biological replicates. Statistical significance was determined by two-way ANOVA with Tukey’s post hoc test (*P < 0.05, ***P < 0.001, ****P < 0.0001).

A ~94 kDa band corresponding to GyrA was detected specifically in Ctr-infected samples (Fig. S3 and Fig. 4). In unexposed cultures, GyrA levels increased from 16 to 24 hpi, in parallel with Hsp60 accumulation (Fig. 4a), correlating with increased needs for negative supercoiling and protein folding assistance. GyrA remained detectable at both 30 and 40 hpi (Fig. S2).

To determine whether Mox exposure altered detectable GyrA levels during mid-cycle, cultures were exposed to Mox initiated at 16 hpi. Mox treatment resulted in a concentration-dependent decrease in detectable GyrA over 6 h, while Hsp60 levels were only modestly affected (Fig. 4b). These results are consistent with a targeted effect on DNA gyrase rather than a total shutdown of gene expression.

To determine whether reduced GyrA levels reflected protein instability, cultures were treated with chloramphenicol at 20 hpi to inhibit *de novo* protein synthesis and monitor decay of pre-existing GyrA. GyrA levels declined gradually over 2–6 h (Fig. 4c), but to a lesser extent than observed with Mox treatment, suggesting that GyrA itself is not inherently highly unstable.

Collectively, these results show that while GyrA is produced and maintained during normal chlamydial development, Mox exposure is associated with a pronounced reduction in detectable GyrA protein that cannot be explained solely by transcriptional changes or basal protein turnover. The relative stability of Hsp60, in contrast to the reduction in GyrA, supports a targeted effect on gyrase that likely precedes broader impacts on protein abundance.

### *Chlamydia* response to Mox involves coordinated changes in GyrA and TopA levels

Because DNA gyrase and TopA jointly maintain supercoiling homeostasis, we asked whether Mox-induced changes in GyrA are accompanied by alterations in TopA abundance. We initially focused on the 22–26 hpi window. Although both proteins were detectable (Fig. S3), the baseline TopA signal in control samples (unexposed to Mox) was relatively low and was further diminished following Mox treatment, preventing reliable quantification during this interval.

**Fig 5:**
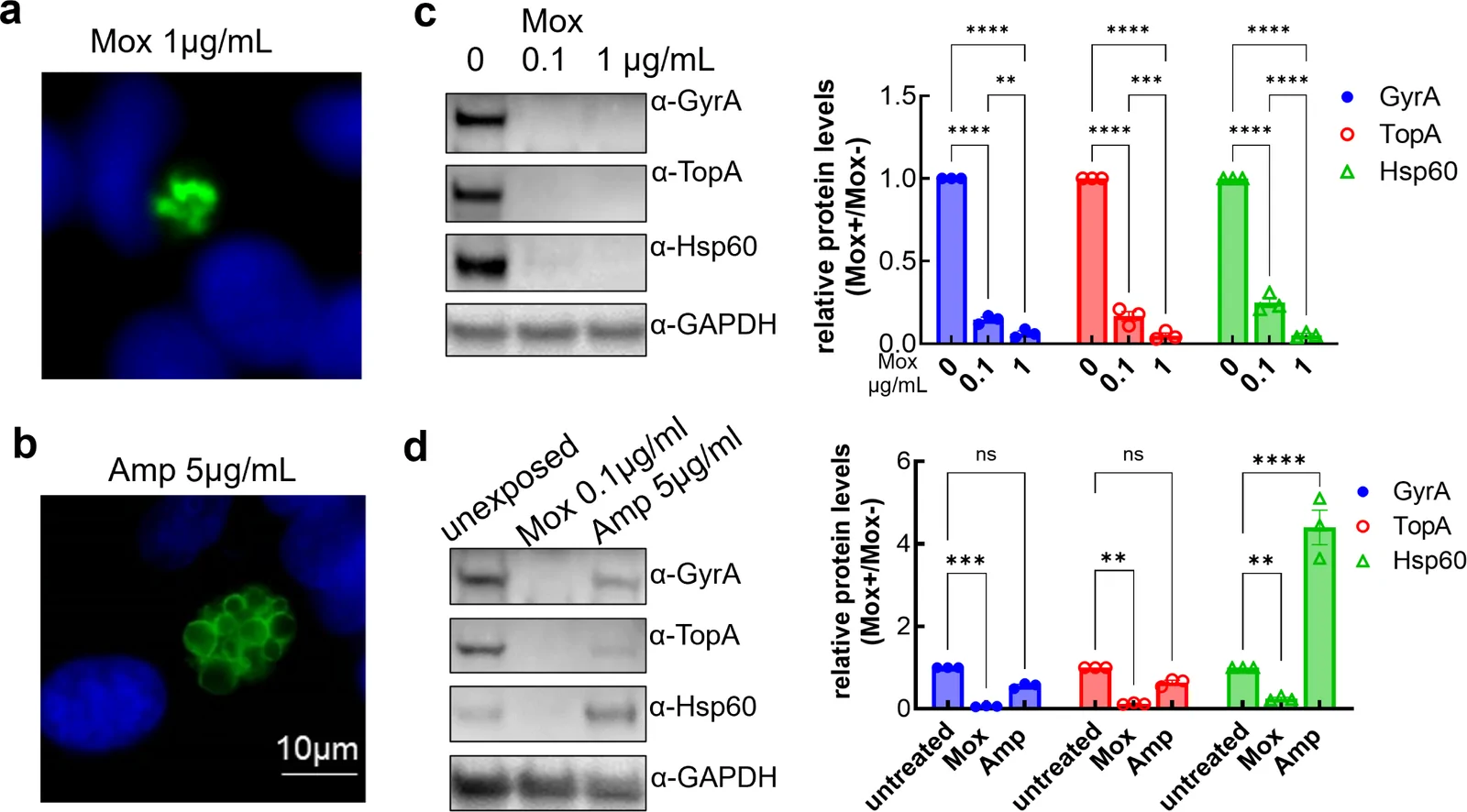
TopA levels decrease following Mox-induced loss of detectable GyrA levels. (**a and b**) Immunofluorescence micrographs of chlamydial inclusions at 40 hpi. L2/434/Bu-infected cells were treated with Mox (**a**) or ampicillin (**b**) starting at 16 hpi, fixed at 40 hpi, and stained for MOMP (green; anti-MOMP) and DNA (blue; DAPI). Scale bar, 10 µm. (**c**) Immunoblot analysis of detectable GyrA, TopA, and Hsp60 in response to Mox. Cultures were treated with Mox (0, 0.1, or 1 µg/mL) starting at 16 hpi and harvested at 40 hpi for analysis. Host GAPDH served as a loading control. (**d**) Quantitative comparison of detectable GyrA, TopA, and Hsp60 protein levels under different stress conditions. Cultures were treated with Mox (at 0.1 µg/mL) or ampicillin (5 μg/mL) starting at 16 hpi and harvested at 40 hpi for analysis. Densitometric analysis of bands was performed using ImageJ. Protein levels were normalized to GAPDH and expressed relative to untreated controls (set to 1). Data represent means ± SD from three independent biological replicates. Statistical significance was determined by one-way ANOVA with Tukey’s post hoc test. **P < 0.01; ***P < 0.001; ***P < 0.0001; ns, not significant.

We, therefore, examined a later time point (40 hpi) when both TopA and GyrA are more readily detected under baseline conditions, evaluating their responses to Mox using immunoblotting. To assess whether these responses are specific to perturbations in DNA topology, extended Mox exposure (16–40 hpi) was compared with ampicillin treatment over the same period as a cell envelope stress control. Ampicillin induced enlarged inclusions containing morphologically aberrant forms distinct from those observed under Mox treatment (Fig. 5a and b). Prolonged Mox exposure resulted in a marked reduction in the detectable immunoblot signals for GyrA, TopA, and Hsp60 relative to those in the unexposed controls (Fig. 5c and d). In contrast, ampicillin treatment caused little decreases in detectable GyrA and TopA signals, while increasing (~4-fold) Hsp60 abundance (Fig. 5d).

These data indicate that a simultaneous drop in detectable GyrA and TopA signals is characteristic of prolonged Mox exposure but is not evident under ampicillin-mediated envelope stress, consistent with a targeted response to the perturbation of DNA topology.

### Supercoiling disruption reprograms promoter-specific transcription

Our findings thus far demonstrate that DNA supercoiling contributes to multiple chlamydial processes and that Mox exposure disrupts this balance. To determine how supercoiling disruption affects virulence-associated loci, we quantified transcript levels of the Hsp60 gene (*groEL1*), its co-transcribed partner *groES*, and the MOMP gene (*ompA*). Given their established roles in pathogenesis and stress adaptation, these genes have been widely studied in models of stress-induced chlamydial persistence (12, 45–47). To evaluate early transcriptional responses, L2/434/Bu cultures were exposed to Mox (0.2 µg/mL) for either 0.5 h (16–16.5 hpi) or 8 h (16–24 hpi), followed by RT-qPCR analysis.

**Fig 6:**
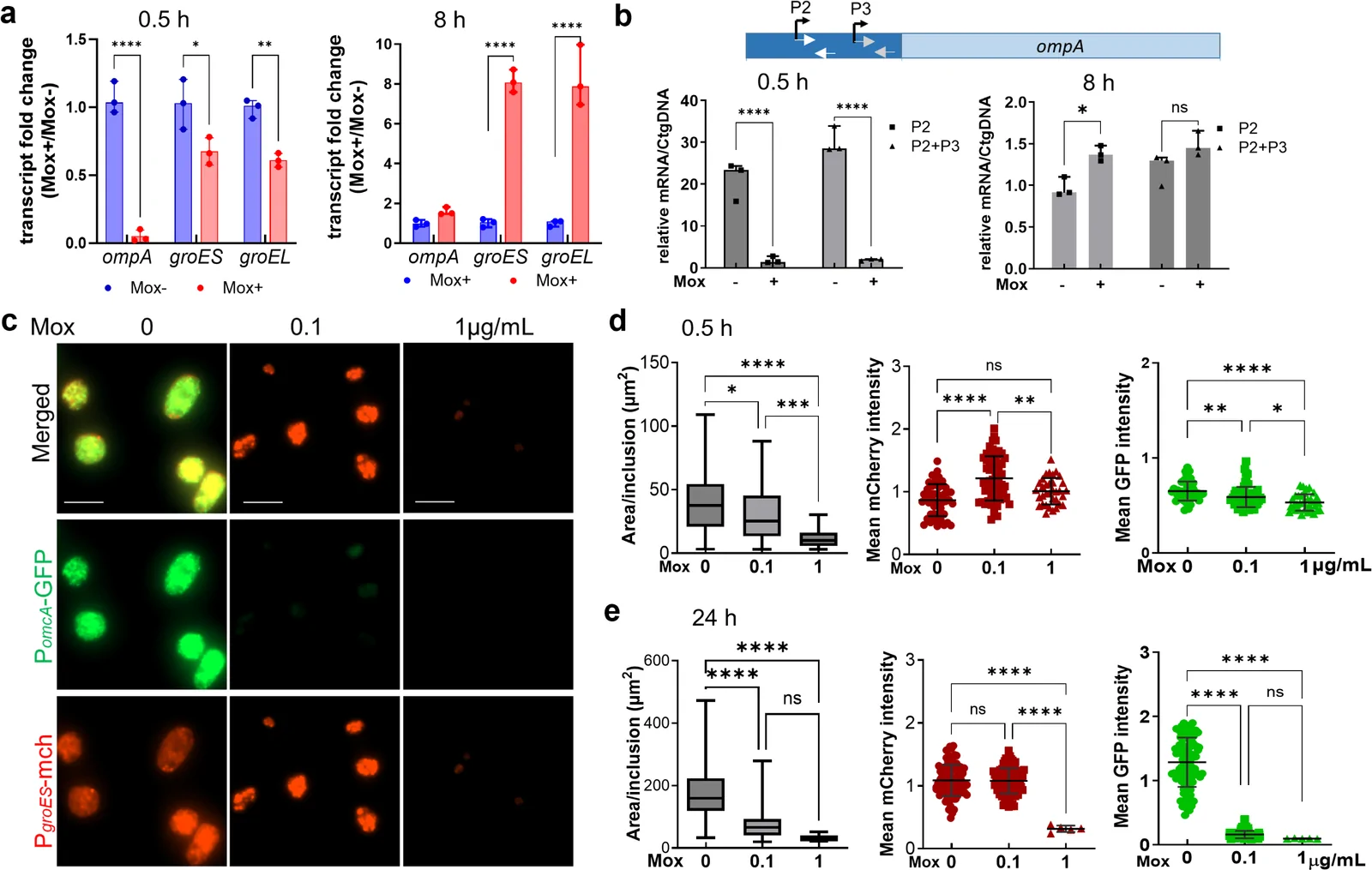
Mox alters promoter-specific transcription using L2/p2TK2-SW2 mCh(GroL2) GFP(OmcAL2). (**a**) Fold changes in *ompA* and *groESL1* transcript levels. Infected cells were treated with Mox (0.2 µg/mL) for 0.5 h (16–16.5 hpi) or 8 h (16–24 hpi). Each gene transcript was independently normalized to its corresponding untreated control sample, which was set to a value of 1. (**b**) Analysis of *ompA* transcripts derived from the P2 promoter or combined P2 + P3 promoters after 0.5 h or 8 h Mox exposure. Arrows indicate primer locations used to amplify P2- or P2 + P3-derived transcripts. (**c**) Live-cell fluorescence imaging of Ctr-infected cells following exposure to increasing Mox concentrations from 16 to 40 hpi. (also see Fig. S5). Signals display fluorescent protein reporters, GFP (green) and mCherry (red), in unfixed cells captured at 40 hpi. Scale bar, 10 µm. (**d and e**) Quantification of inclusion area and mean fluorescence intensity (MFI) after 8 h (16–24 hpi) (**d**) or 24 h (16–40 hpi) (**e**) Mox exposure. For all panels, data represent means ± SD from three independent biological replicates. Statistical significance was determined by ANOVA with Tukey’s post hoc test (*P < 0.05; **P < 0.01; ***P < 0.001; ***P < 0.0001; ns, not significant).

As shown in Fig. 6a, 0.5 h exposure resulted in reduced transcript levels at both *ompA* and *groESL1* loci. However, these responses diverged over time: after 8 h of exposure, *groESL1* transcript levels increased, whereas *ompA* levels were unchanged or remained low. These data are consistent with a shift in transcription toward increased expression of protein-folding machinery under sustained topological stress.

To further assess *ompA* regulation, we examined the activity of its two σ⁶⁶-dependent promoters, P2 and P3 (43), using promoter-specific primers (Table S2). Both P2- and P2 + P3-derived transcripts were reduced within 0.5 h of Mox exposure (Fig. 6b), consistent with the overall decrease in *ompA* transcription (Fig. 6a). At 8 h, a modest recovery was observed only from the P2 promoter (Fig. 6b), suggesting a potential contribution to maintaining residual *ompA* expression under these conditions.

To examine whether the biphasic *groESL1* response reflects temporal changes in promoter activity, we monitored reporter expression alongside chlamydial growth across increasing Mox concentrations using live-cell imaging (Fig. 6c through e and Fig. S5). We used a dual-reporter strain, L2/p2TK2-SW2 mCh(GroL2)GFP(OmcAL2) (48) expressing mCherry red fluorescence protein gene under control of the *groES* promoter (P*_groES_*) and GFP under control of the *omcA* promoter (P*_omcA_*). In unexposed cells, mCherry fluorescence was detectable by 8 hpi, whereas GFP signal appeared by 16 hpi, consistent with early and late promoter activity, respectively. Under Mox exposure, P*_groES_*-driven mCherry signal was maintained or increased with rising Mox concentrations, consistent with the transcriptional trends observed for *groESL1* (Fig. 6a). In contrast, P*_omcA_*-driven GFP signal was undetected or significantly weaker. Only under prolonged, high-concentration exposure (1 µg/mL to 40 hpi) did both reporters decline, suggesting more widespread effects on gene expression under these conditions.

These data indicate that gyrase inhibition preserves stress-responsive transcription from *groESL1*, while suppressing *ompA* and *omcAB* genes, supporting that DNA supercoiling differentially regulates promoter activity and contributes to developmental arrest.

## DISCUSSION

**Fig 7:**
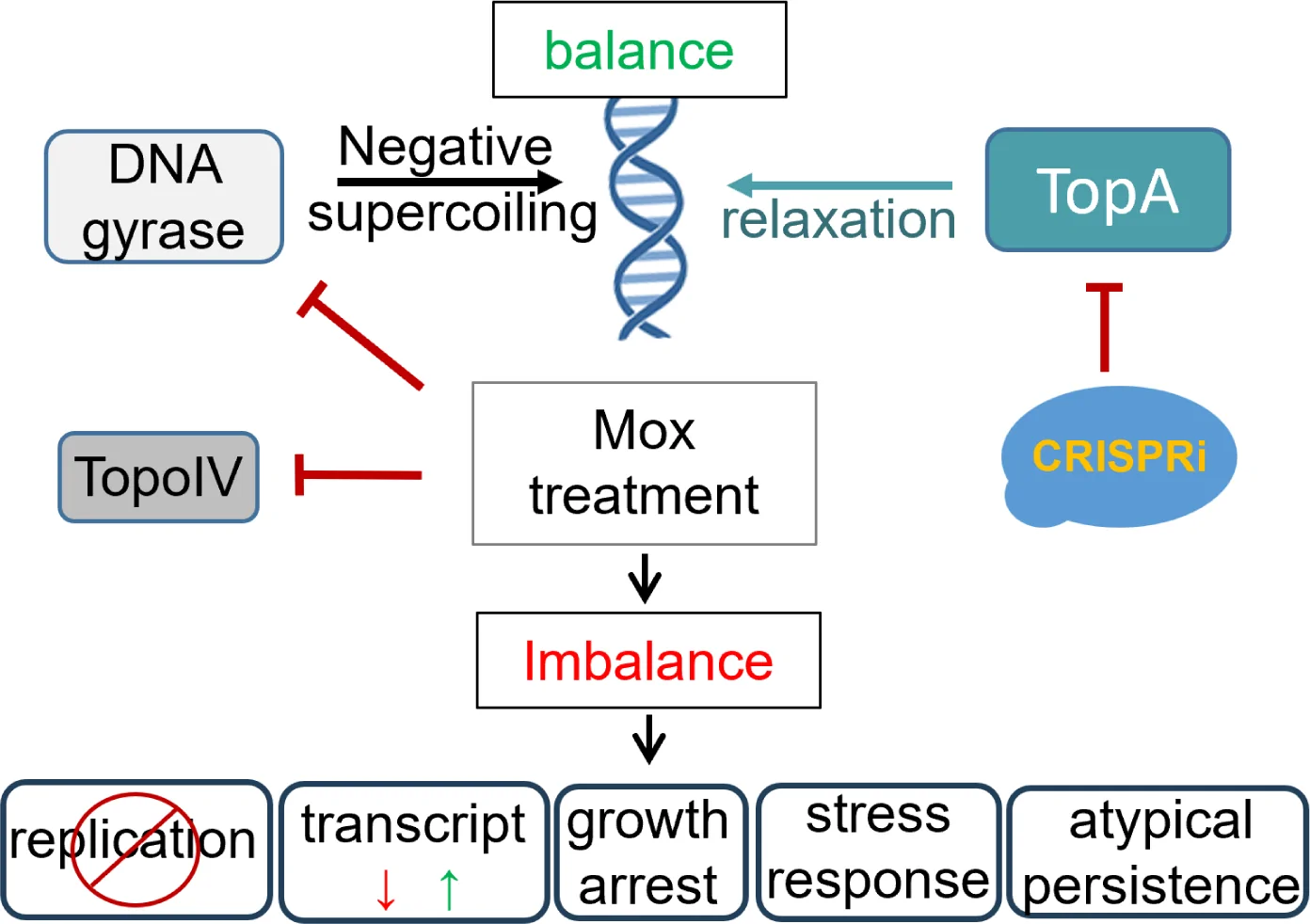
Model of DNA supercoiling control and fluoroquinolone response in *C. trachomatis*. DNA supercoiling homeostasis is maintained by the opposing activities of DNA gyrase, which introduces negative supercoils, and TopA, which relaxes them. Mox targets DNA gyrase (and Topo IV), stabilizing the enzyme–DNA cleavage complex and thereby inhibiting gyrase activity, resulting in reduced negative supercoiling. In contrast, CRISPRi-mediated repression of *topA* reduces DNA relaxation, leading to an increase in negative supercoiling. Disruption of supercoiling homeostasis following Mox-mediated gyrase inhibition creates topological stress that constrains replication and transcription, activates stress responses, and redirects development toward an atypical, replication-limited persistent state.

In these studies, our data indicate that Mox exerts differential effects across developmental stages, inhibits genome replication, reduces functional gyrase pool, and elicits promoter-specific transcriptional responses. Because chlamydial nucleoid architecture and viability depend on DNA supercoiling homeostasis maintained by the opposing activities of DNA gyrase and TopA, perturbation of these enzymes is expected to disrupt topological balance, ultimately altering critical developmental events (Fig. 7). The appearance of enlarged RBs (Fig. 1a and 5a) following Mox exposure is consistent with a disruption of normal developmental progression under conditions that constrain replication and differentiation. Although this non-productive state shares phenotypic features of classical persistence, it is mechanistically distinct because chromosomal replication is compromised (Fig. 2). Accordingly, we refer to it as a replication-limited, noncanonical persistent state.

Prior work establishes DNA supercoiling as a global regulator in bacterial growth and gene expression (25, 49, 50). Our findings extend this concept by suggesting that stage-dependent responses of Ctr to Mox are associated with underlying changes in DNA topology (25–27). This raises the possibility that the physiological impact of gyrase inhibition varies with evolving topological requirements across the chlamydial developmental cycle. In this context, early Mox exposure may interfere with the establishment of negative supercoiling required for the activation of EB chromosomes into a transcription-competent state during primary differentiation. Conversely, mid-cycle organisms (mainly RBs), which experience increased torsional strain from concurrent replication and transcription, may exhibit a greater reliance on gyrase-mediated negative supercoiling. Under condition of impaired gyrase activity, a subset may enter a viable, replication-limited state though the origin and exact properties of this surviving subpopulation remain to be defined. By comparison, the reduced impact of Mox at later stages could reflect chromosomal recondensation and a diminished dependence on gyrase-mediated supercoiling.

Consistent with this model, Mox-mediated inhibition of genome replication (Fig. 2a through c) supports a role for unresolved replication-associated torsional stress in limiting DNA accumulation. Because replication generates positive supercoils that must be resolved by gyrase, its inhibition would be expected to increase torsional strain, impede fork progression, and reduce gDNA levels (35, 51). The pronounced replication defect observed during chlamydial mid-cycle is, therefore, consistent with elevated topological changes during RB proliferation. Moreover, the partial recovery following drug removal suggests that these constrained effects remain reversible within a subset of organisms.

Concurrently, topological perturbation triggered rapid transcriptional responses in topoisomerase genes (Fig. 3), underscoring the tight coupling between DNA topology and its enzymatic regulators. In *Escherichia coli*, gyrase expression relies on a classic negative feedback mechanism where DNA relaxation stimulates transcription to restore basal supercoiling (52, 53). *Chlamydia* exhibits unique temporal and developmental coordination of topoisomerase expression (25). *In vitro* studies suggest that Ctr gyrase promoters are highly sensitive to, and more active on, supercoiled DNA templates (25). However, the marked induction of *gyrAB* transcripts following Mox exposure (Fig. 3) suggests that acute supercoiling perturbations shift transcriptional output, potentially through a compensatory negative feedback loop that operates alongside native developmental programming. In contrast, the minimal changes observed in *topA* and *parCE* transcript levels indicate that topological stress responses during mid-cycle preferentially modulate gyrase expression over a global activation of all topoisomerases. Fully deciphering the underlying regulatory networks that govern this selective expression remains a target for future research.

Protein-level analyses further support DNA gyrase as a principal functional target of Mox (Fig. 4 and 5). Fluoroquinolones trap the covalent gyrase-DNA cleavage complex, preventing re-ligation; this molecular roadblock abruptly arrests replication forks, induces topological stress, and drives lethal chromosomal fragmentation (34, 54–56). Reduced GyrA levels likely reflect a combination of impaired protein synthesis and sequestration in a DNA-bound, insoluble complex that resists recovery under standard lysis conditions (32–34, 54–56). Intriguingly, such phenotypic loss occurs despite the concurrent upregulation of *gyrAB* transcripts (Fig. 2), pointing to post-transcriptional barriers and/or structural entrapment. Impaired translation would further limit production of essential secreted effectors, thereby reducing bacterial fitness. Additionally, cleavage complex-associated secondary processes, such as generation of destructive reactive oxygen species (ROS) (32, 55), may contribute to lethality (32, 54). However, our data suggest that loss of functional GyrA is the dominant driver of Ctr outcomes even when *gyrBA* transcription remains elevated. Because catalytic supercoiling requires an intact GyrA₂GyrB₂ holoenzyme, damage to GyrA subunits is sufficient to compromise enzyme activity even without direct GyrB measurements.

While both DNA gyrase and Topo IV are targets of fluoroquinolones, their relative contributions vary among bacterial taxa, with gyrase typically predominating in Gram-negative species and Topo IV in Gram-positive species (34, 35). Topo IV plays a distinct, critical role in replication by facilitating the decatenation of interlinked daughter chromosomes following replication. While our data indicate a prominent role for gyrase, they do not exclude contributions from Topo IV, particularly under conditions that favor cleavage complex stabilization and replication inhibition. Further investigation is necessary to define the extent and context of Topo IV involvement in Ctr pathogenesis.

Notably, the concurrent decreases in GyrA and TopA following Mox exposure (Fig. 5) suggest coordinated, homeostatic modulation of negative and positive supercoiling likely aimed at preserving topological balance during gyrase impairment. Alternatively, this synchronized reduction could reflect diminished requirements for topoisomerase activity during developmental arrest. Importantly, the absence of this coordinated downregulation under ampicillin-induced cell wall stress demonstrates that Ctr distinguishes topological strain from general envelope stress cues, allowing it to differentially tune topoisomerase regulation in a context-dependent manner.

Beyond topoisomerases, modest changes in supercoiling elicited divergent transcriptional responses, as illustrated by the opposing responses of *ompA* or *omcAB* and *groESL1* (Fig. 6a through e). These findings suggest that DNA supercoiling acts as a regulatory signal coordinating developmental progression and stress responses. Given Ctr’s compact genome, limited transcription factor repertoire, and diverse promoter features (18), promoter architecture likely encodes differential sensitivity to superhelical density, enabling gene-specific responses. Additionally, Mox may engage with other stress-responsive pathways not-yet-identified. Further global transcriptomic analyses will be required to characterize these responses.

Importantly, Mox was used here as a perturbative tool to examine the role of supercoiling in chlamydial development rather than modeling clinical therapy. The disruption of supercoiling homeostasis is associated with a replication-limited, noncanonical persistent state that may support survival under antibiotic stress. Prior reports of similar phenotypic effects induced by other fluoroquinolones (13) are consistent with a conserved, target-mediated contribution of topological stress. Future investigations leveraging direct genetic manipulation of chlamydial topoisomerases will be essential to distinguish primary topological effects from wider stress responses, fully map the chromosomal supercoiling landscape, and exploit these mechanical vulnerabilities for novel antimicrobial strategies.

## MATERIALS AND METHODS

### Chemicals

Antibiotics and chemicals were purchased from MilliporeSigma (St. Louis, MO, USA). Primers were synthesized by Integrated DNA Technologies (Coralville, IA, USA).

### Cell cultures and bacteria growth

HeLa 229 cells (human cervical epithelial carcinoma cells; ATCC CCL-2) were cultured in Dulbecco’s modified Eagle medium (DMEM) supplemented with 1 mM glutamic acid, 5% fetal bovine serum (MilliporeSigma), and 20 μg/mL gentamycin (DMEM-5) at 37°C in an incubator supplied with 5% CO2. Cells were confirmed to be *Mycoplasma* negative by PCR as described previously (57).

Ctr strains used are wild-type L2/434/Bu, L2/P*_ompA_*-GFP (single reporter) (43), and the L2/p2TK2-SW2 mCh(GroL2)GFP(OmcAL2) (dual reporter) (48) (Table S1). When appropriate, ampicillin (5 µg/mL) or spectinomycin (500 µg/mL) were used for selection of transformed Ctr strains. Propagation and purification of wild-type and transformed Ctr organisms were performed as described previously (58). The purified EB pellet was resuspended in sucrose-phosphate-glutamic acid buffer (10 mM sodium phosphate, 220 mM sucrose, and 0.50 mM L-glutamic acid). The EB aliquots were stored at −80°C until use. Aliquots of purified EBs were stored at −80°C until use.

Purified EBs were used to infect cells in culture plates at MOI 0.3 (for phenotype and nucleic acid analyses) or MOI 1 (for Western blot analysis). After centrifugation with a Beckman Coulter model Allegra X-12R centrifuge at 1,600 × *g* for 45 min at 37°C, infected cells were cultured in fresh medium at 37°C for various time periods as indicated in each experimental result. To preserve host cell metabolic defenses and integrity, cycloheximide was not used in all experiments. For Mox treatment, Mox stock solution at 10 mg/mL (in 100% DMSO) was diluted in the corresponding culture medium at the concentration for use. Cultures were incubated in medium with Mox under various conditions as detailed in each experiment. The control medium lacking Mox contains an equal percentage of DMSO (at the final concentration ≤0.1% [vol/vol]). To generate cell envelope stress, ampicillin at 5 µg/mL was added to the culture starting at 16 h and analyzed at 40 hpi. Chloramphenicol at 60 µg/mL was used to inhibit *de novo* protein synthesis in Ctr starting at 20 hpi.

### Antibodies

A rabbit polyclonal anti-GyrA serum was produced with synthesized polypeptide corresponding to residues 818–836 at the C-terminal region of GyrA (Pacific Immunology, Ramona, CA). The final serum was purified by a protein A affinity column. The aliquots with 40% glycerol were stored at −20°C prior to use. The other primary antibodies used are (i) a mouse monoclonal anti-MOMP antibody (L2I-45) specific to the LGV L2 strain (kindly provided by Harlan Caldwell), (ii) a mouse monoclonal anti-Hsp60 antibody (kindly provided by Youxun Zhang), (iii) rabbit polyclonal anti-OmcB (42), (iii) a mouse polyclonal anti-TopA antibody (41), and (iv) a mouse monoclonal antibody to GAPDH (catalog #T5168, MilliporeSigma). The secondary antibodies used are (i) Alexa Fluor 568-conjugated goat anti-mouse IgG (catalog #A11004) from Invitrogen (Carlsbad, CA, USA) and (ii) horseradish peroxidase (HRP)-conjugated goat anti-rabbit IgG (catalog #213204, Abcam) and HRP-conjugated anti-mouse IgG (catalog A0168, MilliporeSigma).

### Microscopy

For live-cell imaging experiments, cells infected with Ctr strains, L2/P*_ompA_*-GFP, and L2/p2TK2-SW2 mCh(GroL2)GFP(OmcAL2) (Table S1) were grown in 96-well culture plates at 37°C. Imaging of actively growing non-fixed cells was obtained directly using an imaging reader Cytation1 (BioTek Instrument) to monitor real-time growth and reporter expression levels. The inclusions and fluorescence intensity profiles were analyzed using Gen5 software.

For indirect IFA, HeLa cell monolayers grown in a glass culture chamber (NUNC) or a coverslip in 24-well culture plate were infected with Ctr to achieve ~30% infection. At various times post infection, the cultures were fixed with 4% (wt/vol) paraformaldehyde dissolved in phosphate-buffered saline (PBS) (pH 7.4) for 15 min at room temperature, permeabilized with 0.1% (vol/vol) Triton X-100 for an additional 15 min, and blocked with 2% (wt/vol) bovine serum albumin in PBS for 30 min. Then, cells were incubated with the indicated primary antibody overnight at 4°C, followed by incubation with Alexa Fluor 488/568-conjugated secondary antibody for 45 min at 37°C. DAPI was used to label DNA. In some experiments, cell images were photographed using an inverted fluorescence microscope (Zeiss Axio Observer D1) and analyzed with AxioVision software, version 4.8.

### Inclusion forming unit assay

To determine the infectious EB number in culture, samples were harvested by scraping three wells of a 96-well plate in SPG and lysed via a single freeze-thaw cycle, serially diluted, and used to infect a fresh HeLa cell monolayer in 96 well plate. After 40 h, cells were fixed with 4% paraformaldehyde and stained with a mouse anti-MOMP antibody, followed by staining with Alexa Fluor 568-conjugated goat anti-mouse IgG. The inclusions were counted using Cytation 1. All experiments were performed three times for three biological replicates. The titers of EBs in cells were converted to inclusion-forming units per milliliter.

### Immunoblotting analysis

Whole cellular lysates were prepared from infected cells by directly lysing in buffer (8 M urea, 10 mM Tris-HCl, pH 8.0) in the absence of DNases. Protein concentration was analyzed by BCA assays (ThermoFisher). Samples in 1×SDS loading buffer (62.5 mM Tris-HCl [pH 6.8], 2% (wt/vol) SDS, 10% glycerol, 2.5% β-mercaptoethanol, and 0.01% (wt/vol) bromophenol blue] were heated at 100°C for 10 min and centrifuged at 15000 rpm for 5 min to obtain protein supernatant. An equal amount of protein was loaded into a single lane of the 4%–15% SDS-polyacrylamide gel (BioRad) for separation. Proteins were transferred onto a PVDF membrane (Millipore) that was then incubated with primary antibody, followed by incubation with horseradish peroxidase-conjugated secondary antibody. The blots were visualized by an enhanced chemiluminescence kit (Kindle Biosciences) and imaged on an Azure c600 imaging system using optimal exposure times. Densitometric quantification was performed on blots using ImageJ, and the results were normalized to relevant controls as indicated in each result.

### Nucleic acid preparation and analysis

Infected cells in 24-well plates were harvested and used to isolate DNA and RNA simultaneously using quick DNA/RNA miniprep kit (catalog # D7001, Zymo Research). Total DNA concentration was quantified using NanoDrop spectrophotometry. Residual DNA in the RNA samples was digested with 20-U RNase-free DNase I in-column for 30 min at room temperature, followed by extensive washing. A total of 2 µg of total RNA per sample was reverse transcribed into cDNA using the high-capacity cDNA reverse transcriptase kit (catalog # 4368814, Applied Biosystems). The Fast SYBR green master mix (ThermoFisher) was used for qPCR assay in 10 µL of reaction mixture on a real-time PCR system (Bio-Rad). Primer pairs used in this study were listed in Fig. S1. Each sample was analyzed in triplicate or quadruplicate in a 96-well plate. A negative control containing no CtgDNA was included. The PCR cycle conditions were as follows: 50°C for 2 min, 95°C for 5 min, 95°C for 3 s, and 60°C for 30 s. The last two steps were repeated for 40 cycles with fluorescence levels detected at the end of each cycle. A standard curve was made from purified L2/434/Bu genomic DNA with serial dilutions for each gene-specific primer pair. Relative transcripts per genome copy were then calculated as the number of transcripts divided by the number of chlamydial genome DNA contents measured with the same primer set.

### Flow cytometry

Infected or mock-infected HeLa cells were detached by trypsinization at 24 hpi, fixed with 2% paraformaldehyde at room temperature for 20 min, and washed with PBS once. Fixed cells resuspended in PBS were directly analyzed in a FACSVantage flow cytometer (BD Biosciences) using the FL-1 (green) channel. Flow cytometry data were recorded for at least 3 × 10^4^ cells per sample. Three biological repeats were examined. Mock infection cells were used as a blank control. Data were analyzed using FlowJo software Version 7.6 (TreeStar Inc) for both the percentage of GFP-expressing cell populations and mean fluorescence intensity.

### Statistical analyses

Data analyses were performed using GraphPad PRISM software as described in each result. Values of *P < 0.05 were considered statistically significant.

## Data Availability

The data that support the findings of this study are available.

## SUPPLEMENTAL MATERIAL

Supplemental tables and figures spectrum.02206-26-s0001.pdf Tables S1 and S2, and Figures S1 to S5

ASM does not own the copyrights to Supplemental Material that may be linked to, or accessed through, an article. The authors have granted ASM a non-exclusive, world-wide license to publish the Supplemental Material files. Please contact the corresponding author directly for reuse.
