# Supplementary material for: Coordinated topoisomerase function shapes the fluoroquinolone response of Chlamydia trachomatis
Source: Microbiol Spectr. 2026 Aug 27;14(10):e02206-26. doi: 10.1128/spectrum.02206-26 (PMC13689479; doi:10.1128/spectrum.02206-26)
Supplement: Supplementary file 1 [file spectrum.02206-26-s0001.pdf]

Supplemental information

Coordinated topoisomerase function shapes fluoroquinolone response in *Chlamydia trachomatis*

Table S1 and Table S2

Figure S1-S5

Table S1 *C. trachomatis* strains and the primers used in this study

C. trachomatis	Description	reference
L2/434/Bu	Bubo isolate from human with lymphogranuloma venereum (LGV).	ATCC VR902B™
L2/P_{ompA}-GFP	*C. trachomatis* carrying an *ompA* P3::GFP reporter plasmid	(1)
L2/P_{EO}	*C. trachomatis* carrying a dual reporter plasmid p2TK2-SW2 mCh(GroL2) GFP(OmcAL2)	(2)

Table S2. Primers used in the present study

Primers	Sequence (5'-3')	Note
rt*CT643*prF	GTTGAATCCCCAGCCAAGATTA	RT-qPCR for *topA* (3)
rt*CT643*prR	CCCTTTTGCAGGAAGATCAACA	
*rtCT660/661*PrF	GAGAATCTTGTTACCAACCTCTAGC	RT-qPCR for *parCE*
*rtct660/661*PrR	GTTCCAAAATGACGTAAGACGC	
rt*ct190/189*PrF	GAGTCACGCTTTATCCATTCGG	RT-qPCR for *gyrAB*
rt*ct190/189*PrR	AGCTCTCCTTCATTTCCTCTTCA	
*topA*_rtF2	CAATCGCATACCAAGCCTTT	RT-qPCR for *topA*
topA rtR2	ACGTAGCCTTTGCCCTTCTTT C	
QP2*ompA*F	GGCGCTTAAGGGCTTCTTCC	RT-qPCR for *ompA* P2-derived transcripts (1)
QP2*ompA*R	AATGGCCTGAGGAATGTCTTGC	
QP2*ompA*/3F	CGACATGTTCTCGATTAAGGCTGC	RT-qPCR for *ompA* P2+P3-derived transcripts
QP2*ompA*/3R	GCGGCACTGCGTCCTAGTTAAA	

1. Cong Y, Gao L, Zhang Y, Xian Y, Hua Z, Elaasar H, Shen L. 2016. Quantifying promoter activity during the developmental cycle of *Chlamydia trachomatis*. Scientific Reports 6:27244.
2. Cortina ME, Ende RJ, Bishop RC, Bayne C, Derré I. 2019. *Chlamydia trachomatis* and *Chlamydia muridarum* spectinomycin resistant vectors and a transcriptional fluorescent reporter to monitor conversion from replicative to infectious bacteria. PLoS One 14:e0217753.
3. Shen L, Gao L, Swoboda AR, Ouellette SP. 2024. Targeted repression of *topA* by CRISPRi reveals a critical function for balanced DNA topoisomerase I activity in the *Chlamydia trachomatis* developmental cycle. mBio 15:e02584-23.

Figure S1

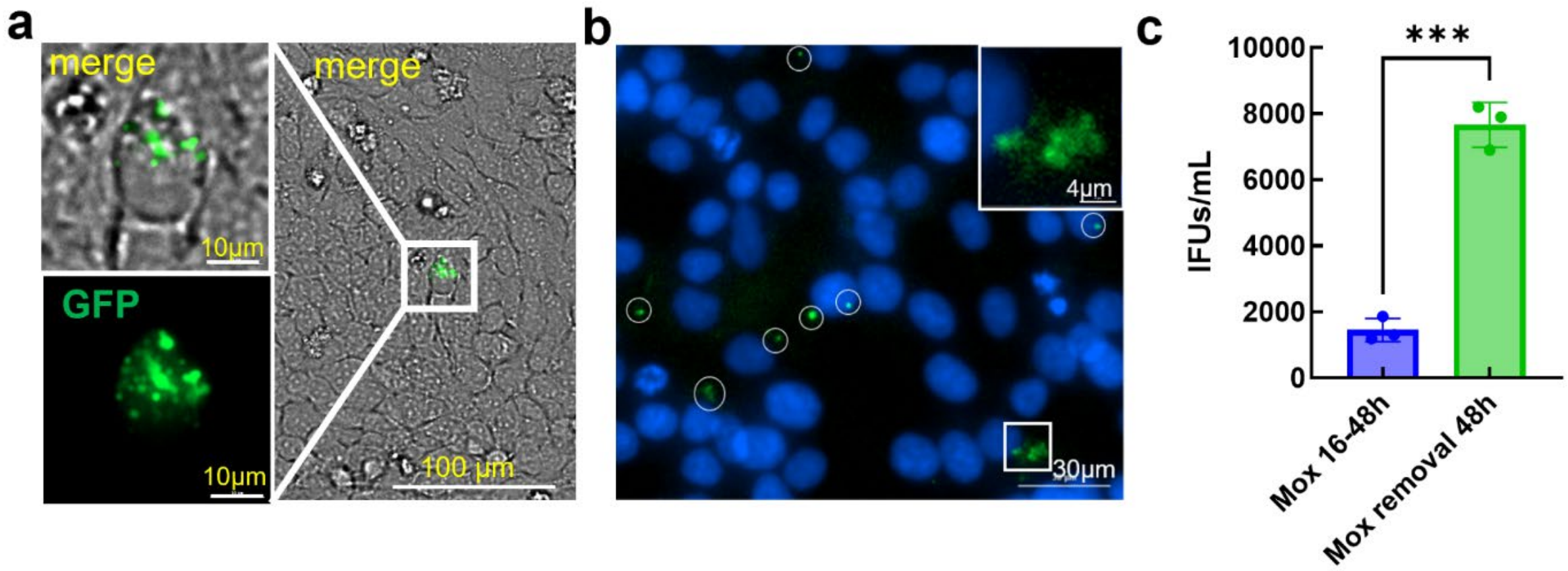

Figure S1 Slow recovery phenotype of Ctr growth following Mox removal. HeLa cells infected with Ctr strain L2/P_{ompA}-GFP at MOI=0.3 were treated with Mox 1µg/mL starting at 16h. At 48h pi, Mox was washed away, and the cultures were incubated in drug-free medium for an additional 48 hpi in the absence of cycloheximide.

- **a.** Expansion of individual inclusion containing GFP expressing Ctr particles (green). Images were captured in unfixed cells following Mox removal for 48 hpi. Inset is enlarged on the left. Scale bars are as indicated.
- **b.** Immunofluorescence micrograph of Ctr infected cells recovered from Mox exposure. Cells fixed at 48 hpi after Mox removal were stained for MOMP (green; anti-MOMP antibody) and DNA (blue; DAPI). Inset shows a magnified inclusion. Small inclusions were circled. Scale bars are as indicated.
- **c.** Enumeration of recoverable EBs using IFU assays. The IFU values represent means ± S D from three biological replicates. Statistical significance was assessed by Welch's t test ***P ≤ 0.001

Figure S2

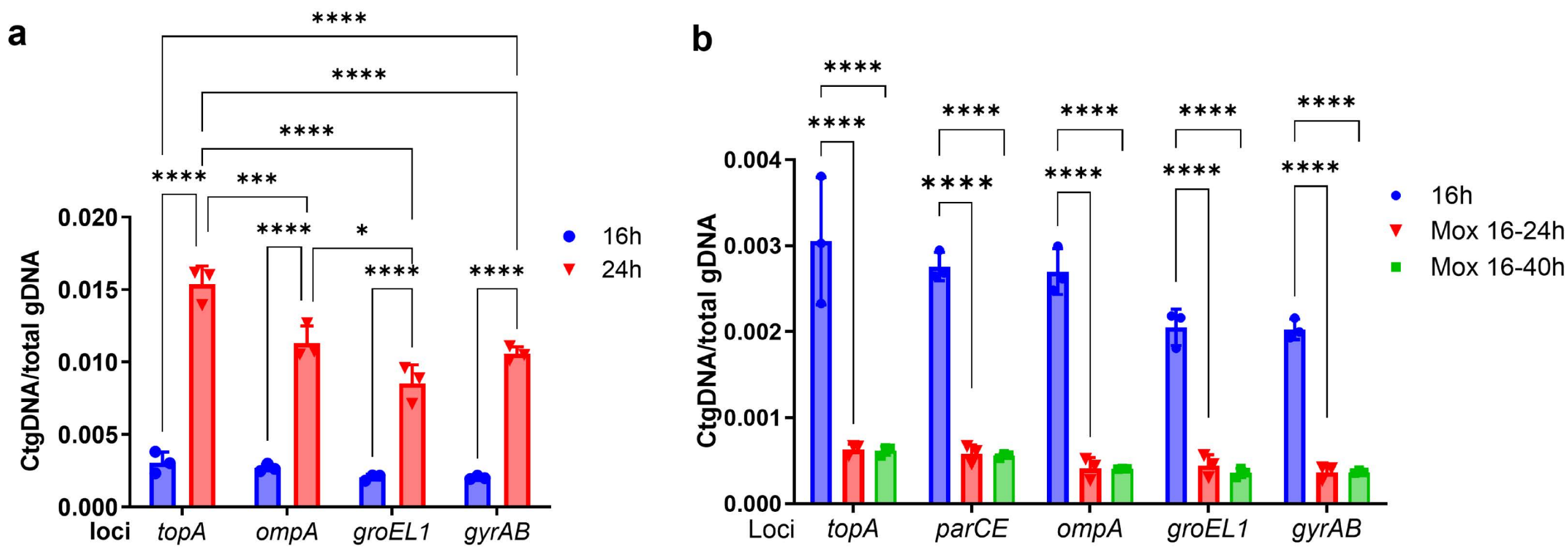

Figure S2. Effect of moxifloxacin on DNA replication. Total genomic DNA (gDNA) extracted from Ctr L2/434/Bu cultures were used to quantify CtgDNA accumulation using qPCR with primers specific to genes as indicated (Table S2).

(a) Analysis of CtgDNA content at 16 hpi and 24 hpi.

(b) Mox induced changes in CtgDNA content from 16 to 24h or 16 to 40h. The levels of CtgDNA were normalized to total gDNA in respective samples. Data represent means ± SDs from three independent experiments.

Statistical significance was determined using two-way ANOVA with the Tukey's post hoc analysis; *P<0.05, ***P ≤ 0.001, ****P ≤ 0.0001.

Figure S3

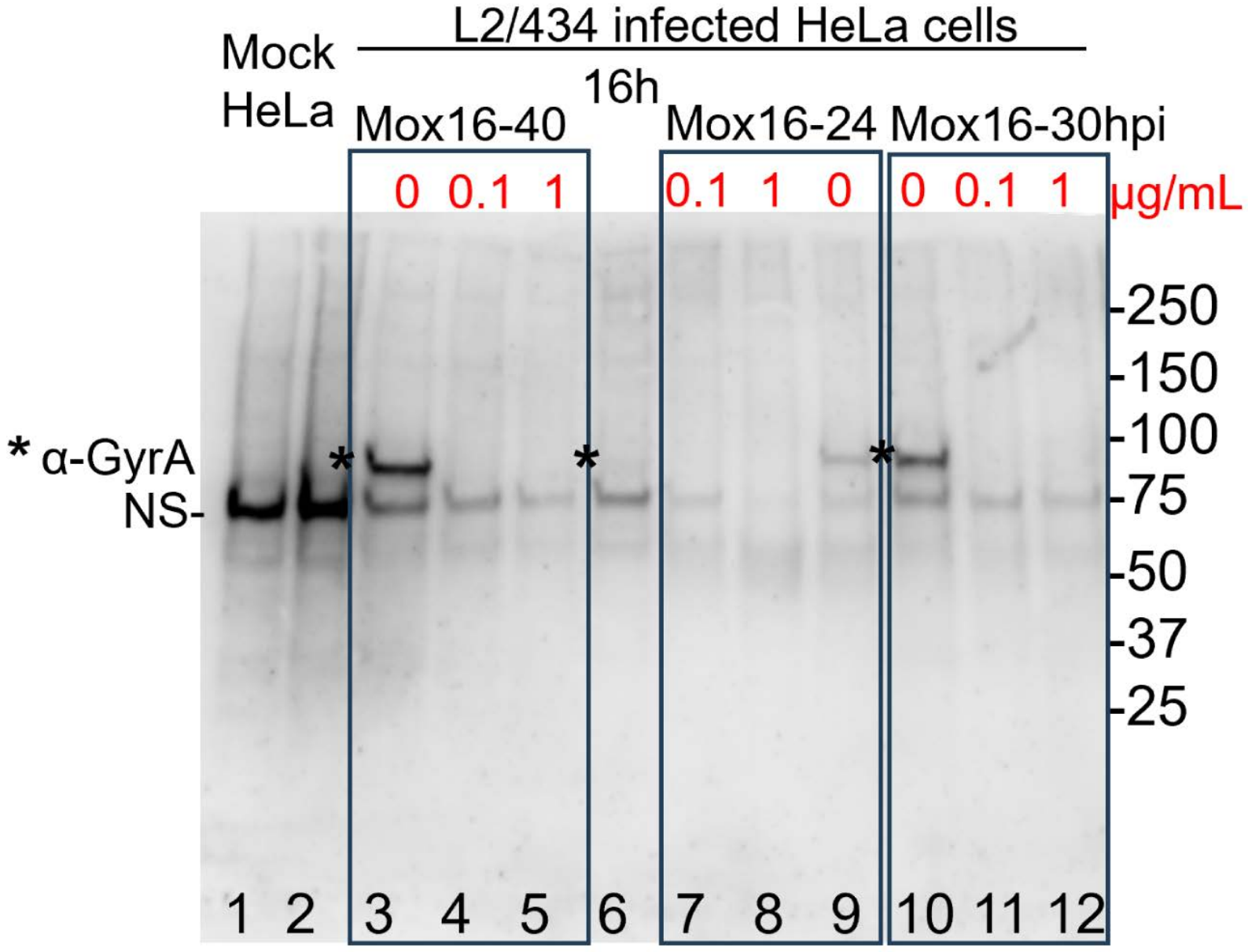

Figure S3. Resprestantative Western blot of lysates from mock-infected and L2/434/Bu infected HeLa cells. Cells were exposed to Mox, at the concentrations as indicated (red font) starting at 16 hpi and incubated for different periods (e.g. 16-40h, 16-24h, or 16-30h) prior to harvesting. Cellular lysates were prepared as described in Methods and probed for GyrA (α-GyrA). The asterisk * shows size of GyrA (~94kDa) that is detected in Ctr samples only, but its detectable levels are significantly decreased in the presence of Mox. NS: a smaller ~75kDa band present in both mock-infected and the Ctr-infected cells likely representing non-specific binding to a host cell component. Note: baseline GyrA signal was detected at 16, 24, 30 and 40 hpi (Lanes 3, 6, 9, and 10). Molecular markers are shown on the left.

Figure S4

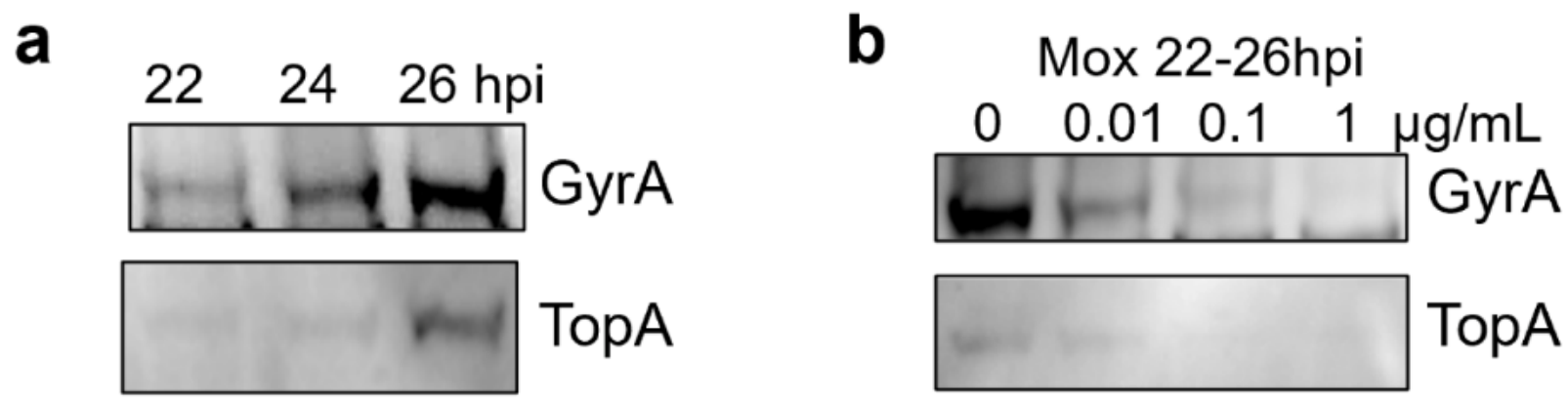

Figure S4 Immunoblot analysis of detectable GyrA and TopA levels in lysates from L2/434/Bu infected HeLa cells.
(**a**) Accumulation of detectable GyrA and TopA in 22-26 h pi window in the absence of Mox.

(**b**) Analysis of detectable TopA and GyrA levels in the presence of Mox at 26h pi. Infected cultures exposed to Mox (at 0, 0.1, 1 µg/mL) initiated at 22 hpi were incubated for an additional 4 h (from 22 to 26h pi) prior to analysis.

Figure S5

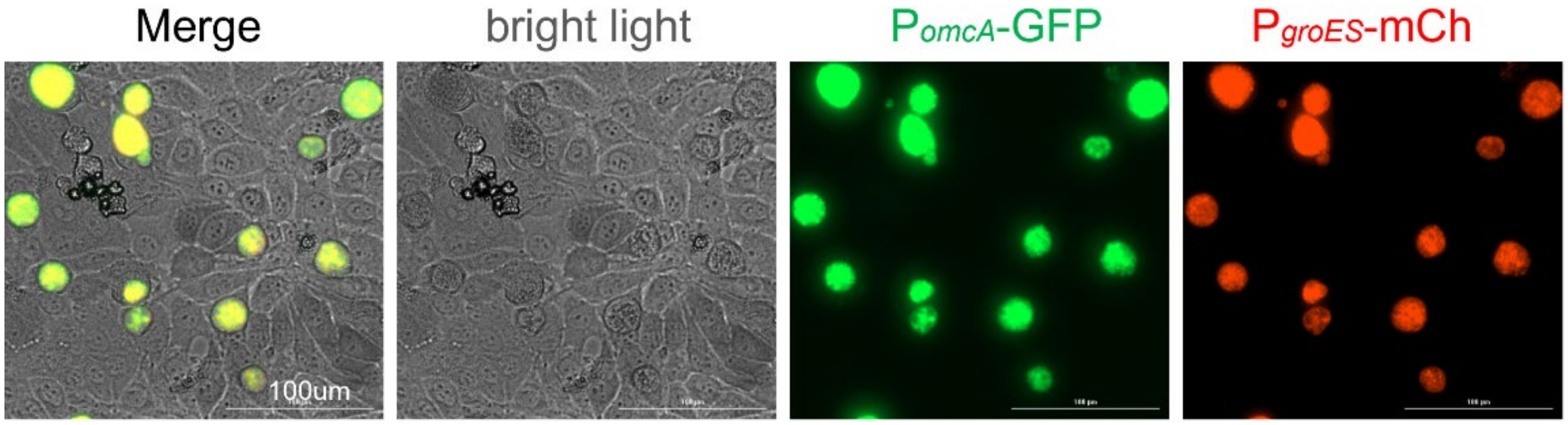

Figure S5. Live-cell fluorescence microscopy of the Ctr reporter strain in HeLa cells. Micrographs captured actively growing, non-fixed cells at 40 hpi. Real-time signals display fluorescent protein reporters GFP (green) and mCherry (red) overlaid with brightfield (bright light) imaging. Scale bar = 100 µm**.**
